# Dendritic Glycerol-Cholesterol Amphiphiles as Drug Delivery Systems: A Comparison between Monomeric and Polymeric Structures

**DOI:** 10.3390/pharmaceutics15102452

**Published:** 2023-10-12

**Authors:** Jocelyn Fernanda Romero, Svenja Herziger, Mariam Cherri, Mathias Dimde, Katharina Achazi, Ehsan Mohammadifar, Rainer Haag

**Affiliations:** Institute of Chemistry and Biochemistry, Freie Universität Berlin, Takustr. 3, 14195 Berlin, Germany; fernandarh@zedat.fu-berlin.de (J.F.R.); svenja.herziger@fu-berlin.de (S.H.); mcherri6@zedat.fu-berlin.de (M.C.); matze111@zedat.fu-berlin.de (M.D.); katharina.achazi@fu-berlin.de (K.A.)

**Keywords:** polyglycerol dendron, cholesterol, RAFT polymerization, polymeric amphiphiles, drug delivery

## Abstract

The application of micelles as drug delivery systems has gained a great deal of attention as a means to overcome the current several drawbacks present in conventional cancer treatments. In this work, we highlight the comparison of polymeric and monomeric amphiphilic systems with a similar hydrophilic–lipophilic balance (HLB) in terms of their biocompatibility, aggregation behavior in aqueous solution, and potential in solubilizing hydrophobic compounds. The polymeric system consists of non-ionic polymeric amphiphiles synthesized via sequential RAFT polymerization of polyglycerol first-generation [G1] dendron methacrylate and cholesterol methacrylate to obtain poly(G1-polyglycerol dendron methacrylate)-block-poly(cholesterol methacrylate) (pG1MA-b-pCMA). The monomeric system is a polyglycerol second-generation [G2] dendron end-capped to a cholesterol unit. Both amphiphiles form spherical micellar aggregations in aqueous solution, with differences in size and the morphology in which hydrophobic molecules can be encapsulated. The polymeric and monomeric micelles showed a low critical micelle concentration (CMC) of 0.2 and 17 μg/mL, respectively. The results of our cytotoxicity assays showed that the polymeric system has significantly higher cell viability compared to that of the monomeric amphiphiles. The polymeric micelles were implemented as drug delivery systems by encapsulation of the hydrophobic small molecule doxorubicin, achieving a loading capacity of 4%. In summary, the results of this study reveal that using cholesterol as a building block for polymer synthesis is a promising method of preparation for efficient drug delivery systems while improving the cell viability of monomeric cholesterol.

## 1. Introduction

Diverse shapes and architectures have been designed as drug delivery systems (DDSs) as an alternative method for anti-cancer drug delivery, aiming to enhance the biocompatibility and tumor targeting that conventional treatments cannot offer [1,2,3]. The ability to transport highly hydrophobic drugs has been another challenge, as delivery systems often show critical issues when interacting with cell membranes [4].

The design of polymeric transport systems from versatile synthetic routes has gained a great deal of attention [5,6], leading to varied architectures such as amphiphilic polymers that are able to self-assemble into polymeric micelles, bilayers, or polymersomes [7,8]. The spontaneous formation of those self-assembled structures relies on the hydrophilic–lipophilic balance (HLB) of the chemical structure, and is observed when the concentration in an aqueous solution is above the critical micelle concentration (CMC) [9].

Recently, a synthetic amphiphilic random copolymer bearing cholesterol moieties (12 wt. %) capable of self-assembly into polymersomes was investigated by Martin et al. [10]. The polymersomes resulted in a ten-fold increase in cellular uptake of dextran into HeLa cells compared to free dextran, improving cellular membrane crossing via endocytosis [10]. The hydroxyl group on the cholesterol structure allows for straightforward synthesis of cholesterol derivatives, with different functional groups usually incorporated into the molecules in two different fashions: either as cholesterol end-capped molecules [11,12] or as chain modified polymers bearing cholesteryl moieties [13,14]. These cholesteryl moieties can be implemented as pendant groups thanks to their high compatibility with anticancer drugs and ability to ameliorate the interaction with the cell membrane as hydrophobic domains of a DDS [4,11,13,15].However, a high concentration of cholesterol-bearing structures shows high cell toxicity; therefore, incorporating the cholesterol with an adjusted HLB ratio can improve the cell viability while enabling the cellular uptake [10]. In this context, a well-defined cholesterol-based “monodisperse” amphiphilic system with precise knowledge about the composition and behavior of biological entities is relevant [16,17]. A great synthetic-route candidate for accomplishing well defined amphiphilic block copolymers is reversible addition–fragmentation chain transfer (RAFT) polymerization [18,19,20,21]. The absence of metal catalysts is an attractive feature of this type of polymerization for biomedical purposes [22,23,24]. In a recent study, a cholesterol end-capped macro-RAFT agent was added to a PNIPAAm backbone to obtain polymeric micelles. The final system did not disrupt red blood cell membranes at concentrations up to 0.25 mg/mL [25].

The prolonged circulation in the blood of micellar systems is mainly a consequence of the physiological behavior of the micelles’ hydrophilic outer shell, which plays a principal role in terms of stability, cellular uptake, and in certain cases reticuloendothelial system (RES) uptake [26,27]. Our group has reported several amphiphilic systems containing polyglycerol (PG) dendrons as hydrophilic parts, which is due to the hydrophilicity and biocompatibility provided to the system by their tree-like structure along with the several terminal hydroxyl groups on the periphery [28,29]. Moreover, their simple chemical structure makes them excellent candidates for incorporation as pendant groups [30]. Recently, Trappmann et al. [31] reported the synthesis of nonionic amphiphilic molecules containing different generations of PG dendrons as hydrophilic parts. These molecules are capable of self-assembly into ring-like, fiber-like, and spherical aggregations for possible application as transport carriers. In another study, nonionic amphiphiles composed of polyglycerol [G2] dendron and single alkyl chains were investigated [32]. The noncytotoxic amphiphilic structures showed superior solubilization properties of hydrophobic molecules than standard excipients used in commercially available surfactant formulations [32].

**Figure 1:**
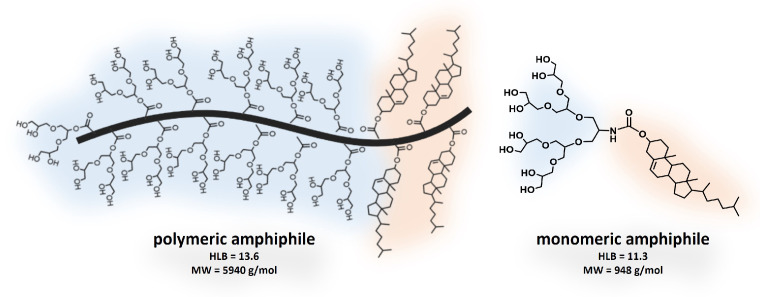
Chemical structure of pG1MAOH-b-pCMA polymeric amphiphiles (**left**) and G2OH-CHOL monomeric amphiphiles (**right**).

The aim of this study is to investigate two analogous tailored micellar structures containing PG dendrons as the hydrophilic part and cholesterol moieties as the hydrophobic part (Figure 1). The first structure (left) is an AB-type block copolymer amphiphile synthesized via RAFT polymerization containing poly(G1-polyglycerol dendron methacrylate) and poly(cholesterol methacrylate) blocks. The second structure (right) is a monomeric amphiphile containing a G2-polyglycerol dendron end-capped with one cholesterol unit. In this study, we compare these two structures based on their solubility, size, shape, and cytotoxicity properties. As a proof of concept, the capability of the polymeric structure as a drug carrier is presented as well.

## 2. Materials and Methods

Chemicals, materials, and methods are described in detail in Appendix A.

### 2.1. Synthesis of Polymeric Amphiphiles

#### 2.1.1. Synthesis of Acetal-Protected G1-Methacrylate Monomer

In a Schlenk flask, G1-OH (5 g, 15.6 mmol) was dissolved in 50 mL DCM. Triethylamine (Et${}_{3}$N, 10 equiv., 21.8 mL) was added to the mixture and cooled down to 0 °C with an ice bath. Methacryloyl chloride (2 equiv., 3 mL) was added dropwise over 30 min with a syringe pump for 1 h. The reaction occurred overnight, letting it reach room temperature. The crude was extracted with DI water three times, dried over MgSO${}_{4}$, and purified by column chromatography (silica, cyclohexane/EtOAc 65/35 *v*/*v*). ${}^{1}$H NMR is shown in Figure S1. Yield: 76%, slightly yellow viscous liquid.

#### 2.1.2. Synthesis of Cholesterol Methacrylate (CMA) Monomer

Cholesterol methacrylate (CMA) was synthesized as reported by Zhou et al. [33]. In a Schlenk flask, cholesterol (5 g) was dissolved in 50 mL THF. In reflux, Et${}_{3}$N (10 equiv., 17.2 mL) was added to the reaction flask at 80 °C. Methacryloyl chloride (2 equiv., 2.5 mL) was added dropwise over 30 min. The mixture was reacted overnight for 20 h and then cooled to room temperature. The crude was concentrated and dissolved in 100 mL of DCM. Liquid–liquid extraction was performed with 200 mL of DI water, HCl (0.5 M), DI water, saturated NaHCO${}_{3}$/H${}_{2}$O (90/10 *v*/*v*), DI water, and brine. The organic phase was dried over MgSO${}_{4}$ and purified by flash column chromatography (silica, cyclohexane/DCM 70/30 *v*/*v*). ${}^{1}$H NMR is shown in Figure S2. Yield: 90%, white powder.

#### 2.1.3. First Step: Synthesis of pG1MA Homopolymer

In a Schlenk flask, the G1MA monomer (1 g, 2.6 mmol) was dissolved in 8 mL of toluene. The solution was deoxygenated by bubbling nitrogen into the solution over 30 min. DDMAT (78.2 mg, 0.21 mmol) and AIBN (0.167 equiv., 6 mg) were quickly added to the reaction flask while bubbling with argon for at least 30 min. The reaction mixture was placed into an oil bath stirring at 70 °C, stirred for 24 h under an argon atmosphere, and quenched with liquid nitrogen, then 2 mL of acetone was added to the flask and the product was purified by dialysis in acetone (2 days, RC membrane, MWCO 3.5 kDa). The final product was characterized by ${}^{1}$H NMR (Figure S3) and GPC (M${}_{n}$ ≌ 5.0 kDa, Đ ≌ 1.5). Yield: 61%, yellow viscous liquid.

#### 2.1.4. Second Step: Synthesis of pG1MA-b-pCMA Block Copolymer

In this step, pG1MA (600 mg) was used as a macro-RAFT agent after previously drying overnight in a Schlenk flask. Toluene (0.6 mL) was added to the reaction flask, followed by the addition of CMA monomer (6 equiv., 380 mg) and AIBN (0.167 equiv., 0.35 mg). The mixture was stirred and purged for at least 30 min. The reaction flask was placed in an oil bath at 70 °C and degassed for over 30 min more. After 20 h of reaction, the reaction mixture was quenched with liquid nitrogen and purified by dialysis in acetone (2 days, RC membrane, MWCO 3.5 kDa). The final product was characterized by ${}^{1}$H NMR (Figure S4) and GPC (M${}_{n}$ ≌ 6.9 kDa, Đ ≌ 1.4). Yield: 41%, yellowish paste.

#### 2.1.5. Synthesis of pG1MAOH-b-pCMA Polymeric Amphiphiles by Deprotection of Dendritic Polyglycerol Units

pG1MA-b-pCHOL polymer (400 mg) was first dissolved in methanol (4 mL). HCl (37%) was added carefully to make up a final acid concentration of 3.7%. The solution was stirred overnight at room temperature and the mixture was purified by dialysis in water (2 days, RC membrane, MWCO 3.5 kDa). The product was lyophilized. Successful deprotection was confirmed by the absence of acetal-group proton signals in the ${}^{1}$H NMR spectrum in D${}_{2}$O (Figure S5). Yield: 81%, white powder.

### 2.2. Synthesis of Monomeric Amphiphiles

#### 2.2.1. Synthesis of Acetal-Protected G2-CHOL

First, cholesterol chloroformate (1.3 g, 2.9 mmol) was dissolved in dry DCM (15 mL). Et${}_{3}$N (0.46 mL, 3.6 mmol) and acetal-protected G2-NH${}_{2}$ (500 mg, 0.7 mmol) were subsequently added to the reaction flask (synthesis described in Appendix B). The mixture was stirred for 18 h at room temperature. After concentration, the crude product was purified by column chromatography (NP, DCM/MeOH 90/10 *v*/*v*). The final product was characterized by ${}^{1}$H NMR (Figure S6). Yield: 61%, colorless oily solid.

#### 2.2.2. Synthesis of G2OH-CHOL Monomeric Amphiphiles by Deprotection of Dendritic Polyglycerol Units

Acetal-protected G2-CHOL (500 mg, 0.5 mmol) was dissolved in methanol (15 mL). After Dowex-H (2 eq/wt.) was added, the reaction mixture was stirred for 3 h at 50 °C. The residue was filtered off and the filtrate was concentrated in vacuo. The crude product was purified by HPLC (RP, MeOH/H${}_{2}$O 15/85 *v*/*v*). Successful deprotection was confirmed by the absence of acetal-group proton signals in the ${}^{1}$H NMR spectrum in D${}_{2}$O (Figure S7). Yield: 86%, light yellow viscous oil.

### 2.3. Preparation of Doxorubicin (Free Base)

DOX·HCl (100 mg, 0.172 mmol) was dissolved in 200 mL of water, then Et${}_{3}$N (1 mL; 7.2 mmol; 42.0 equiv.) was added. The DOX-free base was extracted three times using chloroform (200 mL). The organic phase was dried over Na${}_{2}$SO${}_{4}$, the solvent was removed under reduced pressure, and the product was dried overnight under vacuum. The product was obtained in quantitative yield and stored in the freezer. ESI-MS: *m/z*; calculated: 544.2 g/mol.; found: 544.1 g/mol ([M+H]${}^{+}$).

### 2.4. Micelle Formation and Characterization

A suspension of 5 mg of the respective amphiphiles was prepared in a solution mixture of 0.5 mL of acetone (HPLC grade) and 50 μL of Milli-Q water. The solution was ultrasonicated until no further precipitation was observed. This solution was added dropwise to a vial containing 4.95 mL of Milli-Q water, then acetone was removed under rotary evaporation to obtain micelles with a final concentration of 1 mg/mL. The size and polydispersity index of the micelle solution were recorded by Dynamic Light Scattering (DLS) in low-volume cuvettes at a constant temperature of 37 °C. The ζ-potential was recorded in folded capillary zeta cells at 25 °C. The samples were calibrated for 2 min before each measurement. The critical micelle concentration (CMC) was determined by DLS for pG1MAOH-b-pCMA polymeric micelles and by surface tension for G2OH-CHOL monomeric micelles. DLS studies were performed by observing the derived count rate (in kpcs) at 37 °C for two-folded serial dilutions starting with a concentration of 1 mg/mL of the amphiphiles. The samples were calibrated for 2 min before measurement. Surface tension experiments were recorded in a Dataphysics OCA 20 tensiometer at 25 °C using the pendant drop method. The surface tension of amphiphilic solutions in Milli-Q water was recorded and calculated using the Young–Laplace equation. Samples were vortexed (10 min) and equilibrated 24 h prior to measurement. The surface tension was measured in intervals of 20 s. The experiments were stopped when the surface tension value reached a plateau and did not change for several minutes (in general, after 30–90 min).

### 2.5. Cryo-Tem Imaging

Perforated carbon film-covered microscopical 200 mesh grids (R1/4 batch of Quantifoil, MicroTools GmbH, Jena, Germany) were cleaned with chloroform and hydrophilized by 60 s glow discharging at 10 mA in a Safematic CCU-010 device (Safematic GmbH, Zizers, Switzerland). Subsequently, 4 μL aliquots of the sample solution were applied to the grids. The samples were vitrified by automatic blotting and plunge freezing with an FEI Vitrobot Mark IV (Thermo Fisher Scientific Inc., Waltham, MA, USA) using liquid ethane as a cryogen. The vitrified specimens were transferred to the autoloader of an FEI TALOS ARCTICA electron microscope (Thermo Fisher Scientific Inc., Waltham, MA, USA). This microscope is equipped with a high-brightness field-emission gun (XFEG) operated at an acceleration voltage of 200 kV. Micrographs were acquired on an FEI Falcon 3 direct electron detector (Thermo Fisher Scientific Inc., Waltham, MA, USA) using a 100 μm objective aperture. Graphical analysis of the images was performed using Image J v1.53k.

### 2.6. Encapsulation Studies

Doxorubicin (free base) (DOX) drug was encapsulated in the polymeric micelles by the film method, as described by Prasad et al. [34]. First, a stock solution of DOX was prepared in methanol (C = 1 mg/mL) and 3 mL of drug solution was evaporated under a reduced vacuum forming a film at the bottom of the vial. Then, 20 mL of polymeric micelle solution (C = 1 mg/mL) was added to the vial containing the drug film. The mixture was stirred overnight and filtered through Sephadex G-25 to remove the non-encapsulated drug. The polymeric micelles loaded with DOX were measured by UV-vis at 488 nm. The drug-loading content was calculated from the Lambert–Beer law using a calibration curve of free DOX (Figure S8).

### 2.7. Cytotoxicity Studies

All cell experiments were conducted according to German genetic engineering laws and German biosafety guidelines in the laboratory (safety level 2).

Cell viability was determined using a Cell Counting Kit-8 (CCK-8 Kit, Sigma; Art. 96992) kit according to the manufacturer’s instructions. A549 cells were obtained from Leibniz-Institut DSMZ—Deutsche Sammlung von Mikroorganismen und Zellkulturen GmbH and cultured in Dulbecco’s Modified Eagle Medium (DMEM high glucose GlutaMAX, Gibco; Art. 10566016) supplemented with 10% (*v*/*v*) Fetal Bovine Serum (FBS), 100 U/mL penicillin, and 100 μg/mL streptomycin. A549 cells were routinely cultivated in a 96-well plate at a density of 5 × 10${}^{4}$ cells/mL in 90 μL DMEM medium per well overnight at 37 °C and 5% CO${}_{2}$ until 70% confluency was reached, then subcultured twice a week.

Serial dilutions of the sample in deionized water were prepared; 10 μL of each sample dilution in serial dilutions, including positive (1% SDS) and negative (DMEM) and solvent (H${}_{2}$O) controls, were added and incubated for another 24 h at 37 °C and 5% CO${}_{2}$. For background subtraction, wells containing no cells and only samples were used. After 48 h of incubation, the CCK-8 solution was added (10 μL/well) and the absorbance (450 nm/650 nm) was measured after approximately 3 h incubation of the dye using a Tecan plate reader (SPARK, Tecan Group Ltd., Männedorf, Switzerland). Measurements were performed in technical and biological triplicates. The cell viability was calculated by setting the non-treated control to 100% and the negative control to 0% after subtracting the background signal using Microsoft Excel software v2309. The mean values of all three measurements were plotted and the results were used to determine the half-maximal inhibitory concentration (IC${}_{50}$) using the “log[inhibitor] vs. normalized response (three parameters)” model in GraphPad Prism.

### 2.8. Cellular Uptake Studies

The cells were propagated as described above. For confocal laser scanning microscopy (CLSM), A549 cells (5 × 10${}^{4}$ cells/mL) were seeded in eight-well ibidi slides (ibidi treat) in 270 μL DMEM. After cell attachment for 4–24 h, a post-seeding 30 μL of the compound-containing solution was added for 20 h. Before imaging, the cells were stained with Hoechst 33342 (1 μg/mL), washed with PBS, and covered with fresh cell culture medium (DMEM). Confocal images were taken with a Leica DMI6000CSB SP8 inverted confocal laser scanning microscope (Leica, Wetzlar, Germany) with a 63×/1.4 HC PL APO CS2 oil immersion objective using the manufacturer’s provided LAS X software in sequential mode with the following channel settings: Transmission Ch (grey intensity values), excitation laser line 405 nm, detection of transmitted light (photomultiplier); Ch1 (Hoechst 33342): excitation laser line 405 nm, detection range 408–558 nm (hybrid detector); Ch2 (Doxorubicin free base): excitation laser line 561 nm, detection range 564–670 nm (hybrid detector).

## 3. Results

### 3.1. Synthesis of Polymeric Amphiphiles

A block copolymer of two methacrylate-based monomers was synthesized by sequential RAFT polymerization, with the expectation of AB-type polymerization of the hydrophobic and hydrophilic blocks. As a building block of the hydrophilic part, the acetal-protected polyglycerol [G1] dendron methacrylate (abbreviated as G1MA monomer) was synthesized via the esterification reaction of the acetal-protected polyglycerol [G1] dendron (G1-OH) and methacryloyl chloride. This dendritic precursor was used in its acetal-protected version to avoid any side reactions or intramolecular transfer of the hydroxyl protons. Deprotection of the acetal groups was performed at the end to achieve the hydrophilic block. As a hydrophobic monomer, cholesterol methacrylate (abbreviated CMA monomer) was synthesized as reported by Zhou et al. [33].

**Figure 2:**
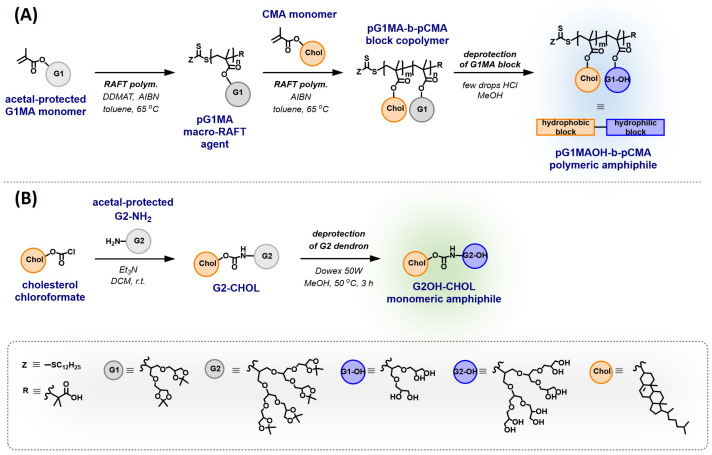
(**A**) Synthetic route of the pG1MA-b-pCMA block copolymer followed by deprotection of the G1MA block to form pG1MAOH-b-pCMA polymeric amphiphiles. (**B**) Synthetic route of the G2-CHOL structure followed by deprotection of the G2 dendron to form G2OH-CHOL monomeric amphiphiles.

The synthetic route overview of the polymeric structure is depicted in Figure 2A. To synthesize the copolymer, the G1MA monomer was polymerized at the first step using 2-(dodecylthiocarbonothioylthio)-2-methylpropionic acid (abbreviated as DDMAT) as a RAFT agent to obtain the pG1MA homopolymer. The pG1MA was purified by dialysis against acetone and used further as a macro-RAFT agent for the polymerization of the CMA monomer in the presence of a small amount of AIBN, which acted as a co-initiator to obtain the pG1MA-b-pCMAdiblock copolymer. In the end, the acid-catalyzed deprotection of the G1MA block was performed afterward in order to obtain the pG1MAOH-b-pCMA polymeric amphiphiles.

**Figure 3:**
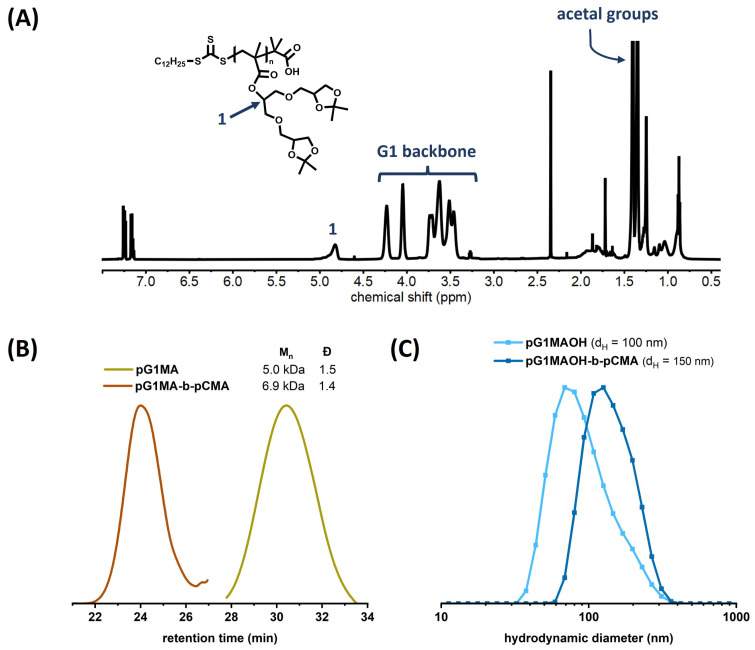
(**A**) ${}^{1}$H NMR of the pG1MA homopolymer. (**B**) GPC chromatograms of pG1MA homopolymer (yellow) and pG1MA-b-pCMA block copolymer (orange), showing an increase in the M${}_{n}$ value from 5.0 to 6.9 kDa. (**C**) Size distributions (% by volume) from DLS for the pG1MAOH-b-pCMA polymeric micelles (dark blue) and pG1MAOH deprotected homopolymer (light blue) in solution.

**Figure A2:**
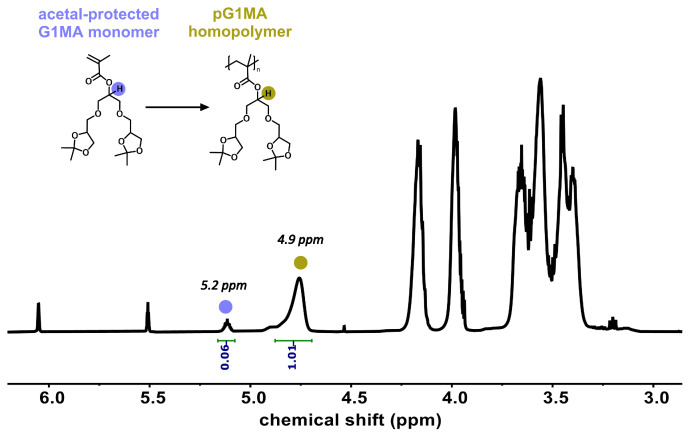
${}^{1}$H NMR spectrum of the crude product obtained from the acetal-protected pG1MA homopolymerization reaction.

The homopolymerization of G1MA was monitored by the signal’s shift of the proton adjacent to the ester group from 5.2 ppm to 4.9 ppm in the ${}^{1}$H NMR spectra of the crude product (Figure A2). A high monomer conversion of 94% was observed from the ${}^{1}$H NMR of the crude product, as described in Appendix C. After purification by dialysis, pG1MA homopolymer was characterized by ${}^{1}$H NMR (Figure 3A) and GPC, the latter showing the targeted molecular weight (M${}_{n}$ ≌ 5.0 kDa) and the narrow polydispersity index (Đ ≌ 1.5) of the produced homopolymer (Figure 3B, yellow line).

pG1MA was used as a macro-RAFT agent in the second step for polymerization of the CMA monomer. Polymerization was carried out with a [macro-RAFT]:[CMA]:[AIBN] molar ratio of 1:6:0.167 in toluene at 65 °C for 20 h and quenched by immersing the reaction flask in liquid nitrogen. The copolymer formation was followed by GPC, observing an increase in the molecular weight from 5 kDa to 6.9 kDa (Figure 3B), resulting in an extent of four cholesterol repeating units to the homopolymer backbone.

The protecting acetal groups from the G1MA block present in the copolymer were hydrolyzed to form the hydrophilic block (Figure 2A). The reaction was acid-catalyzed by dissolving the polymer in methanol, followed by slow dropwise addition of the corresponding amount of hydrochloric acid (37%) to the solution. As a control, deprotection of the pG1MA homopolymer was carried out following the same procedure to obtain hydrophilic pG1MAOH homopolymer.

**Figure 4:**
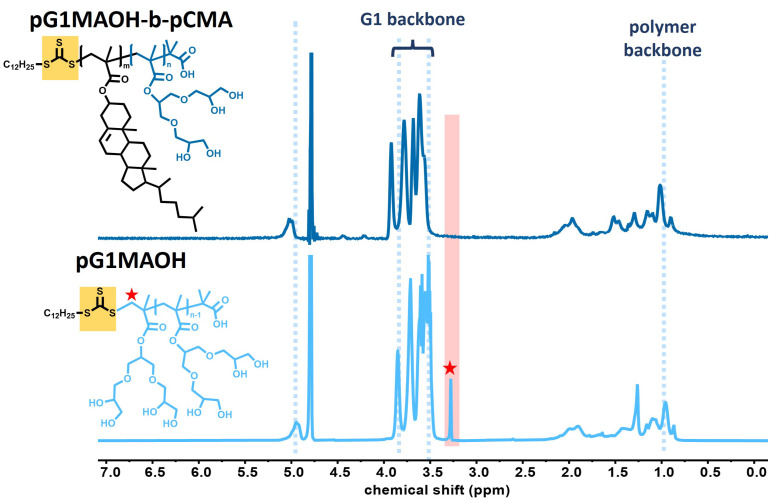
${}^{1}$H NMR in D${}_{2}$O of pG1MAOH-b-pcMA polymeric amphiphiles (**above**) and pG1MAOH control (**below**). The yellow square highlights the thiocarbonothioyl thio group connected to the hydrophobic polycholesterol block (**above**) and to the hydrophilic polyglycerol [G1] dendron block (**below**), leading to the small peak (red star) observed at 3.3 ppm for the latter case.

Figure 4 depicts the ${}^{1}$H NMR for the polymeric amphiphiles (above) compared to the ${}^{1}$H NMR spectra of pG1MAOH control (below) in deuterated water. Deprotection was confirmed in both cases based on the disappearance of the acetal-group proton signal at δ= 1.4 ppm. Regarding the polyglycerol [G1] dendron backbone (3.4–4.2 ppm) and the polymer backbone (1–1.5 ppm), both spectra show a similar shape, with a small shift to higher values in the case of the polymeric amphiphiles (see Figure 4 above). Further, in the chemical structure of the polymeric amphiphile (Figure 4 above) it can be noted that the thiocarbonothioyl thio group (marked in the yellow square) is chemically connected to the polycholesterol block. On the contrary, in the pG1MAOH homopolymer structure (Figure 4 below), the sulfur-containing functional group is directly attached to the hydrophilic polyglycerol [G1] dendron block, leading to the small peak observed at 3.3 ppm (the red star). The absence of this peak in the NMR spectra of the polymeric amphiphile suggests successful copolymer formation, in which the peak at 3.3 ppm should not be present.

### 3.2. Synthesis of Monomeric Amphiphiles

The monomeric amphiphiles consist of the hydrophobic steroidal part of one cholesterol unit in combination with one polyglycerol [G2] dendron unit. For synthesis, the precursor acetal-protected polyglycerol G2-NH${}_{2}$ dendron (abbreviated G2-NH${}_{2}$) was synthesized as the hydrophilic unit following the previously reported synthetic route [35] in five steps (see Appendix B). After purification, the acetal-protected G2-NH${}_{2}$ dendron was reacted in a single step with the commercially available cholesteryl chloroformate in a base-mediated substitution reaction. The acetal-protected structure was deprotected following the procedure reported by Wyzogrodska et al. [35] to obtain the G2OH-CHOL monomeric amphiphiles. An overview of the synthetic route is shown in Figure 2B.

### 3.3. Formation and Characterization of Micelles

The polymeric amphiphiles were subjected to nanoprecipitation in aqueous media to investigate their self-assembly behavior. This behavior occurs when the concentration of the amphiphilic block copolymer exceeds the critical micelle concentration (CMC). Above this concentration, the polymeric structure shows an association due to the desired minimal contact with water forming a stable assembly with low free energy, indicating micellar stability [36]. After nanoprecipitation, it is expected that micelle formation will result from the hydrophobic interactions of the cholesterol blocks forming an inner core, minimizing contact with water molecules, with the outer shell composed of the hydrophilic block of the copolymer. DLS was used to investigate the formation of polymeric micelles at a concentration of 1 mg/mL in water. A monomodal size distribution presented a hydrodynamic diameter of around 150 nm by volume. The ζ-potential (Figure S9) was measured to be −5 mV, corroborating the formation of a non-ionic polymeric micellar system. The CMC of the polymeric micelles was determined by DLS, observing a value of 0.2 μg/mL (Figure S10).

As a control, the pG1MA homopolymer was deprotected following the same procedure to obtain the hydrophilic homopolymer (abbreviated pG1MAOH). The size distribution of the pG1MAOH control aggregates in water was investigated to compare the impact of adding cholesterol units to the polymer structure. DLS results demonstrated a hydrodynamic diameter of around 100 nm for the pG1MAOH control solution in water at 1 mg/mL (Figure 3C). As expected, a small increase in the hydrodynamic diameter was observed in the case of the copolymer amphiphiles when the cholesterol block was included.

On the other hand, monomeric micelles revealed a CMC of 17.0 μg/mL as determined by a surface tension experiment (Figure S11), and presented a monomodal size distribution with a hydrodynamic diameter of around 10 nm (Figure S12). Regarding the hydrophilic–lipophilic balance (HLB) of the systems, monomeric and polymeric amphiphiles showed comparable values of 11.3 and 13.6, respectively, calculated as described in Appendix D. Table 1 summarizes the characterization results of the pG1MAOH-b-pCMA polymeric micelles and the G2OH-CHOL monomeric micelles.

**Figure 5:**
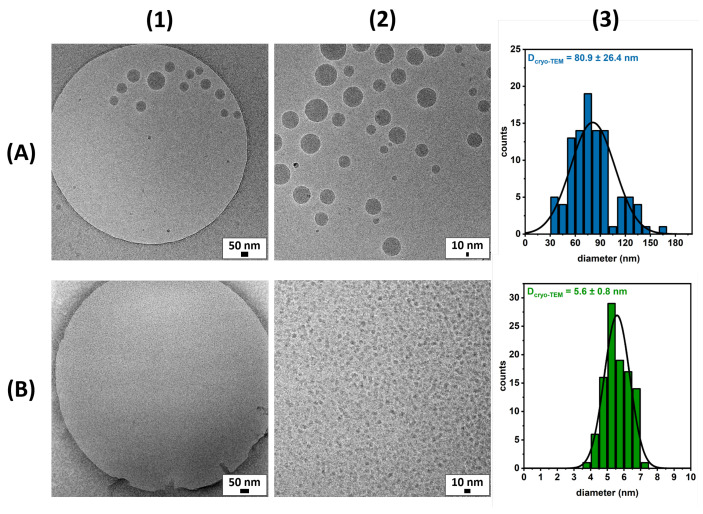
Cryo-TEM micrographs of pG1MAOH-b-pCMA polymeric micelles at (**A1**) 28,000× and (**A2**) 45,000× magnification and G2OH-CHOL monomeric micelles at (**B1**) 58,300× magnification and (**B2**) digital zoom; histograms of 100-micelle size from the analysis of cryo-TEM images of (**A3**) polymeric micelles and (**B3**) monomeric micelles.

The morphology of the micellar solutions was observed by cryogenic Transmission Electronic Microscopy (cryo-TEM). The images showed the self-assembly of the polymeric micelles into spherical aggregations, as shown in Figure 5A1,A2. The diameters of 100 particles were determined and found to be approximately 80 nm (Figure 5A3), corresponding to the typical values of non-ionic polymeric micelles [7]. In the case of the monomeric micelles, small and well-distributed spherical micelles were identified, as shown in Figure 5B1,B2. Similarly, the diameters of 100 particles were determined and found to be around 6 nm (Figure 5B3), showing similar values to other non-ionic dendritic polyglycerol amphiphilic structures [37]. This indicates that the spherical particles are likely composed of a small number of self-assembled amphiphiles [38].

In both cases, the diameters of the particles observed by cryo-TEM were smaller compared to the DLS measurements. This decrease in size can be explained by the differences between the two methods. DLS measures the hydrodynamic diameter is measured, which means that the particles’ hydration shell is considered. On the other hand, the hydration shell is not visualized in cryo-TEM experiments; therefore, smaller diameters were obtained for the micelles.

### 3.4. Cell Viability

**Figure 6:**
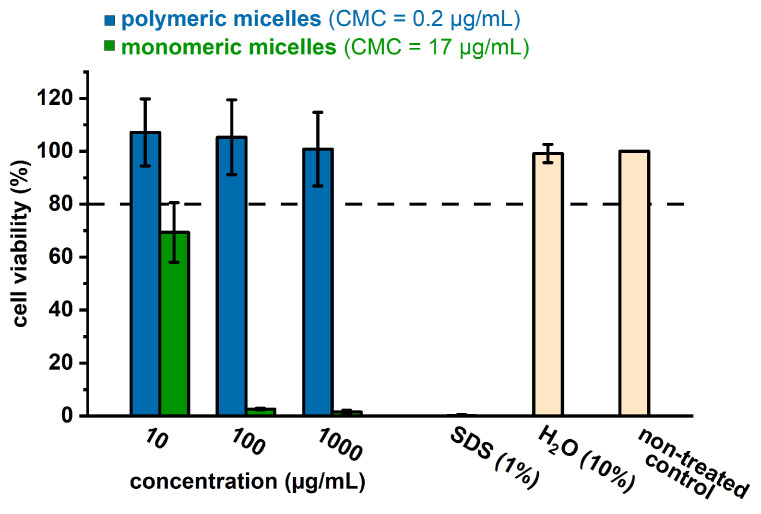
Cell viability of A549 cells treated with polymeric micelles (blue), monomeric micelles (green), and control (light orange). Each bar represents the mean of three independent experiments with the standard deviation.

A preliminary assessment of cytotoxicity (Figure 6) was carried out via CCK-8 Kit assays on A549 cells treated with empty polymeric micelles (blue) and monomeric micelles (green). Ten-fold serial dilutions of the samples were prepared in both cases, starting with 1000 μg/mL micellar solution. As revealed by Figure 6, the monomeric micelles show a severe cytotoxic effect, with less than 10% viable cells at 1000 μg/mL and up to 75% viable cells for a concentration of 10 μg/mL, which is considered very low for employment in biological systems and is below the CMC value (17 μg/mL). According to the literature, the toxic effect of excess cellular free cholesterol results mainly from two protective mechanisms against its accumulation, namely, cholesterol esterification and cellular efflux of cholesterol, and may be due to certain cholesterol-derived oxysterols as well [39]. Thus, the toxic effect of the monomeric micelles can be attributed to the fact that the polyglycerol [G2] dendron end-capped with cholesterol molecules can be inserted into cell membranes following a similar mechanism as free cholesterol [40], resulting in low viability of the treated cells. On the other hand, the polymeric micelles reveal 80% cell viability after 48 h even at high concentrations (1000 μg/mL).

### 3.5. Characterization and In Vitro Studies of DOX-Loaded Polymeric Micelles

**Figure 7:**
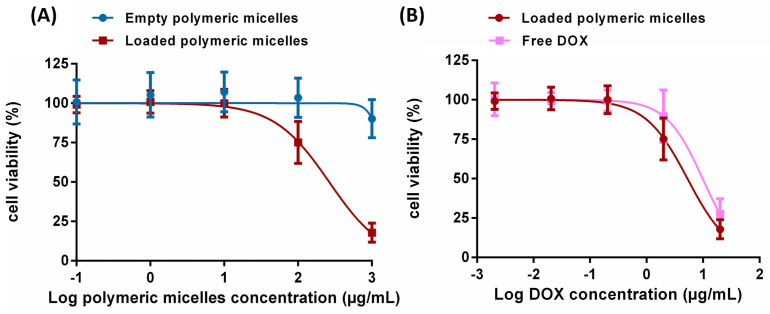
Cell viability of A549 cells after 48 h treatment with (**A**) empty polymeric micelles (blue) and DOX-loaded polymeric micelles (red) based on polymeric micelle concentration, and (**B**) DOX-loaded polymeric micelles (red) and free DOX (pink) based on DOX concentration. Each point represents the mean of three independent experiments with the standard deviation.

The good biocompatibility of the polymeric micelles led us to investigate their efficacy as drug carriers by encapsulating DOX (free base) as a drug model. Here, a hydrophobic interaction between the cholesterol block and the drug was expected to be the driving force of drug loading. The micelles were loaded with 20 wt% of the drug and purified before characterization. The loading capacity (LC) and encapsulation efficiency (EE) of the micelles were calculated as stated in Appendix E. The polymeric micellar system showed a loading capacity of 4% and an encapsulation efficiency of 14%. DLS characterization of the DOX-loaded micellar solution revealed a monomodal particle distribution (Figure S13) with a slightly smaller hydrodynamic diameter of loaded micelles (140 nm) compared to empty micelles (around 150 nm). The shrinking behavior of the encapsulated system could be due to the hydrophobic host–drug interaction. Further, cryo-TEM micrographs of the DOX-loaded polymeric micelles were investigated, where a spherical micellar shape was again observed (Figure S14). These results show that there is no significant impact on size and morphology after the encapsulation of doxorubicin in the polymeric micelles. The viability of A549 cells treated with DOX-loaded polymeric micelles was compared to non-loaded polymeric micelles and free DOX (Figure S15) to inspect the possibility of DOX being released from the micelles and promoting cell death. Therefore, the IC${}_{50}$ values were determined through plot fitting using a nonlinear regression model (inhibitor) vs. the normalized response, first for the non-loaded and DOX-loaded micelles based on the polymeric micelles concentration (Figure 7A), and then for the DOX-loaded micelles and free DOX based on DOX concentration (Figure 7B). Based on the micelles concentration, the IC${}_{50}$ of the empty micelles was 1555.0 μg/mL, in comparison to 264.5 μg/mL for the loaded ones, indicating that the DOX-containing micelles are indeed more toxic than the empty micelles. Based on the free DOX concentration, the DOX-loaded in the micelles had an IC${}_{50}$ of 5.3 μg/mL, while the IC${}_{50}$ of free DOX was 9.8 μg/mL. This implies that DOX might be effectively encapsulated, solubilized, and sustainably released from the micelles and that the encapsulation does not affect the activity of the drug or its effect on the cells. The IC${}_{50}$ values are summarized in Table 2.

**Figure 8:**
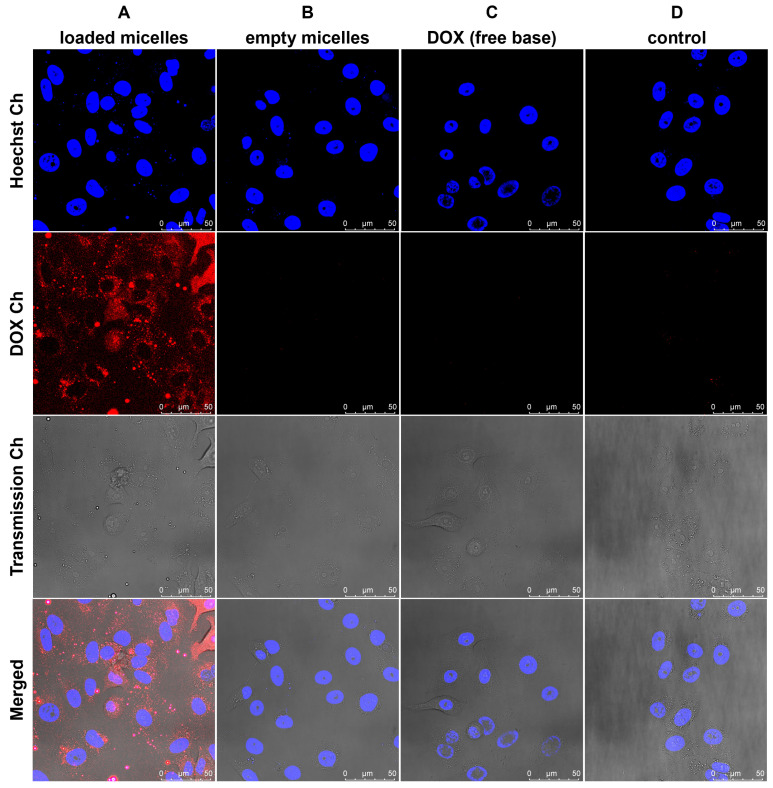
Confocal Laser Scanning Microscopy (CLSM) images of A549 cells treated for 20 h with (**A**) DOX-loaded polymeric micelles (100 μg/mL polymeric micelles + 2% DOX formulation), (**B**) empty polymeric micelles (100 μg/mL), and (**C**) free DOX (2 μg/mL). (**D**) Non-treated cells served as a control. Blue shows Hoechst staining in the nuclei, while red shows DOX. For all images, the scale bar represents 5 μm.

Confocal laser scanning microscopy (CLSM) was used to monitor the cellular uptake of living A549 cells after 20 h treatment with the DOX-loaded and empty polymeric micelles as well as the uptake of free DOX after incubation. The red fluorescence observed in the cell nucleus treated with DOX-loaded polymeric micelles (Figure 8A) suggests that free doxorubicin was released from the polymeric micelles, taken up inside the cells, and mainly distributed in the cytoplasm and within the nuclei, as the polymeric micelles are too large (d${}_{H}$ = 145 nm) to enter the nucleus. Additionally, the red fluorescence of cells treated with DOX-loaded polymeric micelles (Figure 8A) is qualitatively more intense than free DOX (Figure 8C) at the same tested concentration of DOX (2%), suggesting enhanced uptake by A459 cells and an improvement in the bioavailability of doxorubicin (free base).

## 4. Discussion

Herein, we have employed RAFT polymerization to synthesize well-defined non-ionic polymeric amphiphiles composed of polyglycerol dendrons as the hydrophilic block and polycholesterol as the hydrophobic block. The amphiphiles are capable of self-assembly behavior, as confirmed by cryo-TEM images. The micelle diameter was around 80 nm as measured by cryo-TEM, and the hydrodynamic diameter was 150 nm as measured by DLS. In addition, a polyglycerol–cholesterol monomeric structure was synthesized with a similar hydrophilic–lipophilic balance on the part of the polymeric amphiphiles. Their morphology was observed by cryo-TEM, revealing smaller spherical micelles of 6 nm in diameter, while the hydrodynamic size was shown to be 10 nm by DLS measurement. The CMC of both systems was detected in a typical micellar range of 0.1–10 mg/L. As a proof of concept, the polymeric micelles were tested as possible drug carriers, using doxorubicin as a hydrophobic drug model. The obtained loading capacity was 4%, while the morphology and the hydrodynamic size remained similar to those of empty carriers.

The cytotoxicity studies of the polymeric micelles using the A549 cell line suggest good biocompatibility up to relatively high concentrations (1000 μg/mL). In comparison, the monomeric micelles revealed a severe cytotoxic effect at the same concentration, suggesting that monomeric micelles may penetrate the cell membrane by following a mechanism similar to free cholesterol. On the other hand, the presented polymeric micelles combine a low CMC (0.2 μg/mL) and a highly tested biocompatible concentration (1000 μg/mL), suggesting a broad safe window of micellar concentrations for application as a biocompatible drug delivery system. The cellular uptake of the DOX-loaded polymeric micelles was examined via confocal laser scanning microscopy. Our observations indicated that the carriers are taken up efficiently by A549 cells, showing a possible sustained release of doxorubicin, as indicated by the accumulation of DOX in the cell nucleus.

All of these properties, together with the incorporation of cholesterol as pendant groups in the design of a polymeric structure, suggest a promising method of preparation for efficient drug delivery systems while improving the cell toxicity of the monomeric cholesterol structure.

## Figures and Tables

**Table 1:** Characterization of polymeric and monomeric micelles.

Micelles System	MW (g/mol)	CMC (μg/mL)	$d_{H}\;\pm \;\mathrm{SD}$ (nm)	HLB ^1^
polymeric	5940	0.2 ^2^	145 ± 7	13.6
monomeric	948	17.0 ^3^	10 ± 1	11.3

MW: molecular weight. CMC: critical micelle concentration. d${}_{H}$: hydrodynamic size. SD: standard deviation. HLB: hydrophilic-lipophilic balance. ^1^ Calculated with Equation (A5). ^2^ Determined by DLS. ^3^ Determined by surface tension.

**Table 2:** IC${}_{50}$ values based on the polymeric micelles concentration and DOX concentration for A549 cells.

	IC${}_{50}$ (μg/mL Polymeric Micelles)	IC${}_{50}$ (μg/mL DOX)
empty micelles	1555.0	−
DOX-loaded micelles	264.5	5.3
free DOX	−	9.8

**Figure A1:**
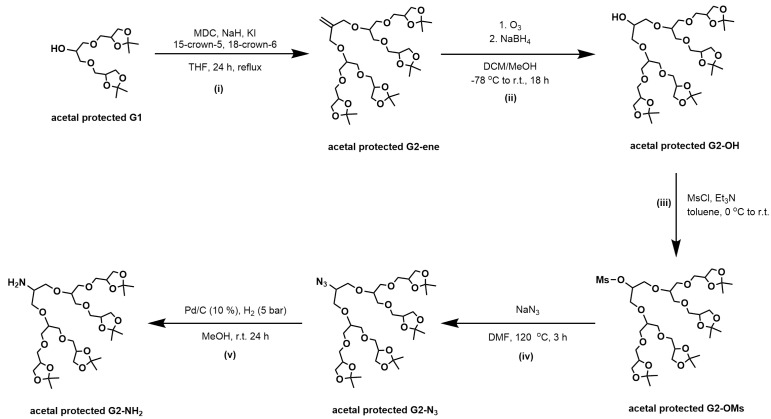
Synthesis of acetal-protected G2-NH${}_{2}$ precursor in five steps: (i) synthesis of acetal-protected G2-ene, (ii) synthesis of acetal-protected G2-OH, (iii) synthesis of acetal-protected G2-OMs, (iv) synthesis of acetal-protected G2-N${}_{3}$, and (v) synthesis of acetal-protected G2-NH${}_{2}$.

## Data Availability

All data are contained within the article or Supplementary Materials.

## Supplementary Materials

The following supporting information can be downloaded at: https://www.mdpi.com/article/10.3390/pharmaceutics15102452/s1. Figure S1: ${}^{1}$H NMR spectrum of the acetal-protected G1 methacrylate (G1MA) monomer. Figure S2: ${}^{1}$H NMR spectrum of the cholesterol methacrylate (CMA) monomer. Figure S3: ${}^{1}$H NMR spectrum of the pG1MA homopolymer. Figure S4: ${}^{1}$H NMR spectrum of the pG1MA-b-pCMA block copolymer. Figure S5: ${}^{1}$H NMR spectrum of the pG1MAOH-b-pCMA polymeric amphiphiles. Figure S6: ${}^{1}$H NMR spectrum of the acetal-protected G2-CHOL. Figure S7: ${}^{1}$H NMR spectrum of the G2OH-CHOL monomeric amphiphiles. Figure S8: (A) U*v*/*v*is measurements at different concentrations of DOX free base and (B) calibration curve obtained at 488 nm. Figure S9: Zeta potential of pG1MAOH-b-pCMA polymeric micelles. Figure S10: CMC determination of pG1MAOH-b-pCMA polymeric micelles. Figure S11: CMC determination of G2OH-CHOL monomeric micelles. Figure S12: Size distributions (% by volume) from DLS for the G2OH-CHOL monomeric micelles. Figure S13: Size distributions (% by volume) from DLS for the DOX@pG1MAOH-b-pCMA. Figure S14: Cryo-TEM micrographs of DOX-loaded polymeric micelles at 28,000× magnification (left) and 45,000× magnification (right). Figure S15: Cell viability of A549 cells treated with polymeric micelles (blue), DOX-loaded polymeric micelles (red), and free DOX (pink). Each bar represents the mean of three independent experiments with standard deviation.

## Abbreviations

The following abbreviations are used in this manuscript:

CMC	Critical Micelle Concentration
DDS	Drug Delivery System
DOX	Doxorubicin
EE	Encapsulation Efficiency
LC	Loading Capacity
MW	Molecular Weight
RAFT	Reversible Addition–Fragmentation Chain Transfer

## Appendix A. Chemicals, Materials, and Methods

### Appendix A.1. Chemicals and Materials

Acetal-protected polyglycerol [G1] dendron (G1-OH) was synthesized as reported by Alpugan et al. [41] and purified by High-Pressure Liquid Chromatography (HPLC). Acetal-protected second-generation polyglycerol dendron-NH${}_{2}$ (G2-NH${}_{2}$) was synthesized and purified as described in Appendix B. All commercially available compounds were used as received without further purification. All reactions were carried out with extra-dry over molecular sieve conditions solvents. Solvents for work-up procedures and column chromatography (i.e., DCM, acetone, chloroform, hexane, EtOAc) were of analytical grade. Millipore water (Milli-Q) was obtained from a Merck Millipore Milli-Q Integral System. All polymers were purified by dialysis using pretreated regenerated cellulose (RC) tubing (MWCO 3.5 kDa) Spectra/Por 7^®^ dialysis membranes.

### Appendix A.2. Methods

Nuclear Magnetic Resonance (NMR) spectra were recorded on either a Bruker AVANCE III 500 (Bruker Corporation, Billerica, MA, USA), a Jeol ECP 500 (JEOL GmbH, Freising, Germany), or a Bruker AVANCE III 700 (Bruker Corporation). Chemical shifts are reported in δ (ppm) and referenced to the respective deuterated solvents.

The Gel Permeation Chromatography (GPC) system was equipped with a Shimadzu Prominenze-i LC-2030 model, a model Shimadzu RID-20A refractive index detector (Nakagyo-ku, Kyōto, Japan), and a three-column set of PPS PolarSil analytical columns (5 μm particle size, 1000 Å, 300 Å, and 100 Å 8 × 300 mm columns). Calibration of the column system was carried out using monodispersed linear polystyrene standards. The system was equilibrated at 40 °C using a mixture of DMF, LiBr (0.3 wt%), and AcOH (0.6 wt%) as both polymer solvent and eluent. The flow rate was set to 1 mL/min. The polymer solutions were prepared at a known concentration (5–8 mg/mL) and an injection volume of 100 μL was used. The solutions were allowed to swell for at least 30 min before filtration with 0.2 μm RC syringe filters.

Dynamic Light Scattering (DLS) characterization was performed using a Malvern Zetasizer Ultra (Malvern Instruments Limited, Malvern, UK) equipped with a 10 mW He-Ne laser operating at a wavelength of 632.8 nm. The scattered light was detected using the backscattering setting at an angle of 173° (NIBS, noninvasive backscatter). The measurements were carried out in disposable plastic cuvettes at a constant temperature of 37 °C in triplicates. All samples were calibrated for 2 min before the experiment was performed.

UV-Vis Spectroscopy was recorded with a LAMBDA 365 U*v*/*v*is Spectrophotometer using disposable plastic cells in a wavelength range of 400–1100 nm.

## Appendix B. Synthesis of Acetal-Protected G2-NH_2_ Precursor

**(i) Synthesis of acetal-protected G2-ene.** Acetal-protected G1-OH (2.0 g, 6.2 mmol) was dissolved in 20 mL dry THF under an argon atmosphere. After the addition of NaH (60% in mineral oil, 620 mg, 15.5 mmol) and catalytic amounts of 15-crown-5, the reaction solution was stirred for 1.5 h at 40 °C. Afterwards, methallyl dichloride (MDC, 310 mg, 2.5 mmol) and catalytic amounts of potassium iodide (KI) and 18-crown-6 were added and the reaction solution was refluxed for 16 h. The reaction was monitored by TLC. After all starting material had been consumed, the reaction solution was cooled to room temperature and quenched with water. The aqueous layer was extracted with DCM and the combined organic layers were washed with brine and dried over Na${}_{2}$SO${}_{4}$. The crude product was concentrated and purified by column chromatography (silica, hexane/i-propanol 92/8 *v*/*v*). Yield: 74%, light yellow oil. ${}^{1}$H NMR: (CD${}_{3}$OD, 400 MHz) δ = 5.21–5.17 (m, 2H), 4.28–4.20 (m, 4H), 4.16 (d, J = 9.2 Hz, 3H), 4.05 (dd, J = 8.1, 6.6 Hz, 5H), 3.78–3.46 (m, 22H), 1.38 (s, 12H), 1.33 (s, 12H) ppm. ${}^{13}$C NMR: (CD${}_{3}$OD, 101 MHz) δ = 144.77, 114.64, 110.50, 79.90, 78.55, 76.25, 76.15, 73.40, 72.90, 72.56, 72.43, 72.35, 71.66, 71.16, 67.76, 67.59, 49.00, 27.11, 25.69 ppm. IR (ATR) ν: 2985, 2932, 2870, 1654, 1456, 1370, 1253, 1212, 1075, 1050, 974, 974, 916, 841 cm ${}^{-1}$. MS (ESI): m/z = calculated 715.4 ([M+Na]${}^{+}$) 731.4 ([M+K]${}^{+}$); measured 715.4 ([M+Na]${}^{+}$) 731.4 ([M+K]${}^{+}$).

**(ii) Synthesis of acetal-protected G2-OH.** Acetal-protected G2-ene (830 mg, 1.1 mmol) was dissolved in dry DCM (8 mL) and dry methanol (8 mL). Ozone was bubbled through the reaction solution at −78 °C. After the reaction mixture turned blue, excess ozone was removed by flushing the solution with oxygen and stirring vigorously. Next, NaBH${}_{4}$ (416 mg, 11 mmol) was added and the reaction solution was stirred for 16 h while warming to room temperature. The reaction solution was quenched with saturated NH${}_{4}$Cl solution at 0 °C followed by stirring for 1 h. The aqueous layer was extracted with DCM. The combined organic layers were washed with water and dried over Na${}_{2}$SO${}_{4}$ before being concentrated in vacuo. Yield: 90%, colorless oil. ${}^{1}$H NMR: (CD${}_{3}$OD, 400 MHz) δ = 4.29–4.20 (m, 4H), 4.02–4.08 (m, 4H), 3.87–3.79 (m, 1H), 3.77–3.46 (m, 24H), 1.39 (s, 12H), 1.33 (s, 12H) ppm. ${}^{13}$C NMR: (CD${}_{3}$OD, 101 MHz) δ = 110.51, 110.45, 79.87, 76.12, 73.42, 72.44, 71.15, 70.88, 67.72, 67.58, 27.11, 25.68 ppm. IR (ATR) ν: 3487, 2985, 2872, 1456, 1370, 1254, 1212, 1050, 974, 841, 734 cm${}^{-1}$. MS (ESI): m/z = calculated 719.4 ([M+Na]${}^{+}$) 735.4 ([M+K]${}^{+}$); measured 719.4 ([M+Na]${}^{+}$) 735.3 ([M+K]${}^{+}$).

**(iii) Synthesis of acetal-protected G2-OMs.** Acetal-protected G2-OH (600 mg, 0.86 mmol) was dissolved in dry toluene (8 mL) at 0 °C. After the subsequent addition of Et${}_{3}$N (0.14 mL, 1.03 mmol) and MsCl (0.073 mL, 0.95 mmol), the reaction was stirred for 18 h while warming to room temperature. The reaction was monitored by TLC. After all starting material had been consumed, the reaction mixture was filtered and the filtrate was concentrated in vacuo. The crude product was used without further purification. Yield: 84%, colorless oil.

**(iv) Synthesis of acetal-protected G2-N_3_.** Acetal-protected G2-OMs (491 mg, 0.63 mmol) was dissolved in dry DMF (3 mL) under an argon atmosphere. After NaN${}_{3}$ (206 mg, 3.1 mmol) was added, the reaction mixture was stirred for 4 h at 120 °C. The reaction mixture was filtered and concentrated in vacuo. The crude product was purified by column chromatography (silica, hexane/EtOAc 1/6 *v*/*v*). Yield: 81%, colorless oil. ${}^{1}$H NMR: (CD${}_{3}$OD, 400 MHz) δ = 4.29–4.21 (m, 4H), 4.02–4.08 (m, 4H), 3.79–3.50 (m, 27H), 1.39 (s, 12H), 1.33 (s, 12H) ppm. ${}^{13}$C NMR: (CD${}_{3}$OD, 101 MHz) δ = 110.46, 80.02, 79.83, 76.23, 76.12, 73.42, 72.48, 72.17, 71.18, 67.74, 67.57, 62.25, 27.12, 25.71 ppm. IR (ATR) ν: 2985, 2933, 2872, 2096, 1738, 1456, 1370, 1252, 1212, 1057, 1049, 974, 840 cm${}^{-1}$. MS (ESI): m/z = calculated 744.4 ([M+Na]${}^{+}$) 760.4 ([M+K]${}^{+}$); measured 744.4 ([M+Na]${}^{+}$) 760.4 ([M+K]${}^{+}$).

**(v) Synthesis of acetal-protected G2-NH_2_.** Acetal-protected G2-N${}_{3}$ (1.7 g, 2.2 mmol) was dissolved in methanol (50 mL) in a reactor vessel. After cautious addition of 10 wt% Pd/C (400 mg, 0.4 mmol of Pd), the reactor was closed and 5 bar of hydrogen was added. The reaction solution was stirred overnight and monitored by TLC. After all starting material had been consumed, the reaction solution was filtered through a celite pad and a syringe filter (0.45 nM) before being concentrated in vacuo. Yield: 99%, colorless oil. ${}^{1}$H NMR: (DMSO, 400 MHz) δ = 4.19–4.11 (m, 4H), 4.00-3.94 (m, 4H), 3.64–3.56 (m, 6H), 3.53–3.32 (m, 20H), 1.31 (s, 12H), 1.26 (s, 12H) ppm. ${}^{13}$C NMR: (DMSO, 101 MHz) δ = 108.45, 77.84, 74.40, 74.27, 71.79, 70.73, 66.13, 65.96, 50.66, 48.61, 26.62, 25.40 ppm. IR (ATR) ν: 3384, 2984, 2870, 1680, 1456, 1370, 1253, 1211, 1074, 1050, 974, 841 cm${}^{-1}$. MS (ESI): m/z = calculated 696.4 ([M+Na]${}^{+}$) 718.3 ([M+Na]${}^{+}$) 734.3 ([M+K]${}^{+}$); measured 696.4 ([M+K]${}^{+}$) 718.4 ([M+Na]${}^{+}$) 734.4 ([M+K]${}^{+}$).

## Appendix C. Calculation of Monomer Conversion

The monomer conversion of pG1MA homopolymerization was calculated by

(A1) $$p\;=\;\frac{\int H_{\mathrm{polymer}}}{\int H_{\mathrm{monomer}}+\int H_{\mathrm{polymer}}},$$

where ∫H is the integral of the proton adjacent to the ester functional group. In Figure A2, the proton corresponding to the monomer is marked in purple at 5.2 ppm and the proton corresponding to the polymer is marked in yellow at 4.9 ppm.

## Appendix D. Calculation of the Degree of Polymerization, Molecular Weights, and HLB Values

The molecular weights of the pG1MA homopolymer and pG1MA-b-pCMA copolymer were determined via GPC (Figure 3C).

The degree of polymerization of the hydrophilic part (X${}_{n,\;H}$) was calculated from the GPC results of the pG1MA homopolymer (M${}_{n,\;\mathrm{pG}1\mathrm{MA}}$) as follows:

(A2) $$X_{n,\;H}\;=\;\frac{M_{n,\;\mathrm{pG}1\mathrm{MA}}}{MW_{G1\mathrm{MA}}},$$

where MW${}_{G1\mathrm{MA}}$ is the molecular weight of the G1MA monomer.

The degree of polymerization of the lipophilic part (X${}_{n,\;L}$) was calculated from the difference in the number-average molecular weights of the copolymer and the homopolymer (ΔM${}_{n}$):

(A3) $$X_{n,\;L}\;=\;\frac{\Delta M_{n}}{MW_{\mathrm{CMA}}}\;=\;\frac{M_{n,\;\mathrm{pG}1\mathrm{MA}-b-\mathrm{pCMA}}-M_{n,\;\mathrm{pG}1\mathrm{MA}}}{MW_{\mathrm{CMA}}},$$

where MW${}_{\mathrm{CMA}}$ is the molecular weight of the CMA monomer.

The theoretical molecular weight of the pG1MAOH deprotected homopolymer and the pG1MAOH-b-pCMA polymeric amphiphiles were calculated assuming the complete removal of acetal groups in the G1MA backbone using the following equation:

(A4) $$M_{n}\;=\;M_{n,\;\mathrm{protected}}-2\cdot p_{H}\cdot m_{\mathrm{acetal}},$$

where M${}_{n,\;\mathrm{protected}}$ is the molecular weight before deprotection, p${}_{H}$ is the degree of polymerization of the hydrophilic part, and m${}_{\mathrm{acetal}}$ is the acetal mass loss when deprotection occurs. The second term refers to the presence of two acetal groups per G1MA repeating unit.

The Hydrophilic–Lipophilic Balance (HLB) of non-ionic amphiphiles can be calculated with Griffin’s method [42] using Equation (A5):

(A5) $$\mathrm{HLB}\;=\;20\cdot \frac{MW_{H}}{M_{n}},$$

where MW${}_{H}$ is the molecular weight of the hydrophilic part in the amphiphilic structure and M${}_{n}$ is the molecular weight of the complete structure.

**Table A1:** Summary of molecular weights and HLB values.

Compound	M${}_{n}$ (g/mol)	p${}_{H}$	p${}_{L}$	MW${}_{H}$ (g/mol)	HLB ^1^
pG1MA	5000	12	−	−	−
pG1MA-b-pCMA	6900	12	4	−	−
pG1MAOH-b-pCMA	5940 ^2^	12	4	4040	13.6
pG1MAOH	4040 ^2^	12	−	4040	−
G2OH-CHOL	948	−	−	536	11.3

MW: molecular weight. p${}_{H}$: degree of polymerization of the hydrophilic part. p${}_{L}$: degree of polymerization of the lipophilic part. HLB: hydrophilic–lipophilic balance. ^1^ Calculated with Equation (A5). ^2^ Theoretical M${}_{n}$ values calculated with Equation (A4).

## Appendix E. Loading Capacity (LC) and Encapsulation Efficiency (EE) Calculation

The loading capacity (LC) and encapsulation efficiency (EE) were respectively calculated using

(A6) $$LC\left(\%\right)\;=\;\frac{m_{\mathrm{encapsulated}\;\mathrm{DOX}}}{m_{\mathrm{amphiphile}}}\cdot 100,$$

(A7) $$EE\left(\%\right)\;=\;\frac{m_{\mathrm{encapsulated}\;\mathrm{DOX}}}{m_{\mathrm{initial}\;\mathrm{DOX}\;\mathrm{added}}}\cdot 100.$$
