# Supplementary material for: Dendritic Glycerol-Cholesterol Amphiphiles as Drug Delivery Systems: A Comparison between Monomeric and Polymeric Structures
Source: Pharmaceutics. 2023 Oct 12;15(10):2452. doi: 10.3390/pharmaceutics15102452 (PMC10610414; doi:10.3390/pharmaceutics15102452)
Supplement: Supplementary file 1 [file pharmaceutics-15-02452-s001.zip › pharmaceutics-2613738-supplementary.pdf]

Supplementary Materials: Dendritic Glycerol-Cholesterol Amphiphiles as Drug Delivery Systems: A Comparison between Monomeric and Polymeric Structures

Jocelyn Fernanda Romero, Svenja Herziger, Mariam Cherri, Mathias Dimde, Katharina Achazi, Ehsan Mohammadifar*, and Rainer Haag*

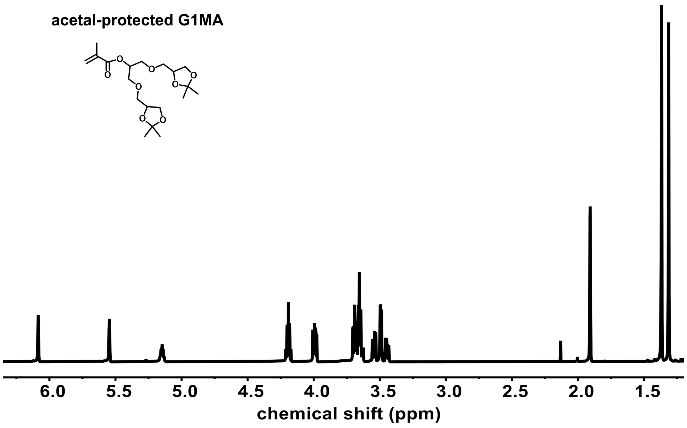

Figure S1. ^{1}H NMR spectrum of the acetal-protected G1 methacrylate (G1MA) monomer.

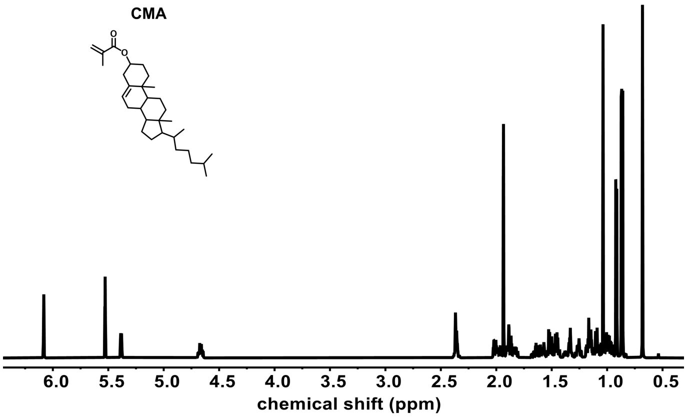

Figure S2. ^{1}H NMR spectrum of the cholesterol methacrylate (CMA) monomer.

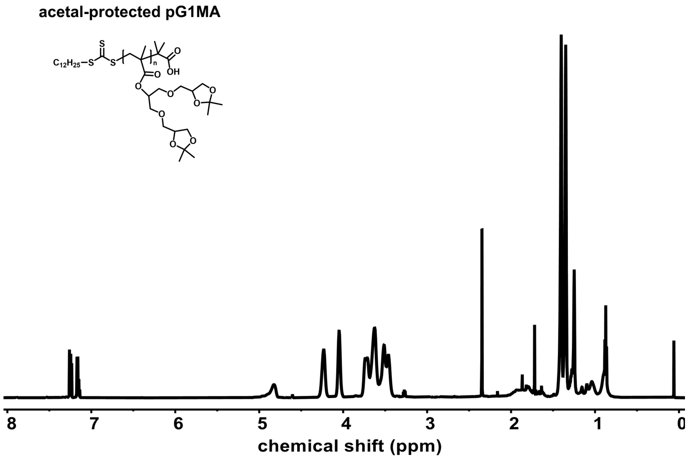

Figure S3. ^{1}H NMR spectrum of the pG1MA homopolymer.

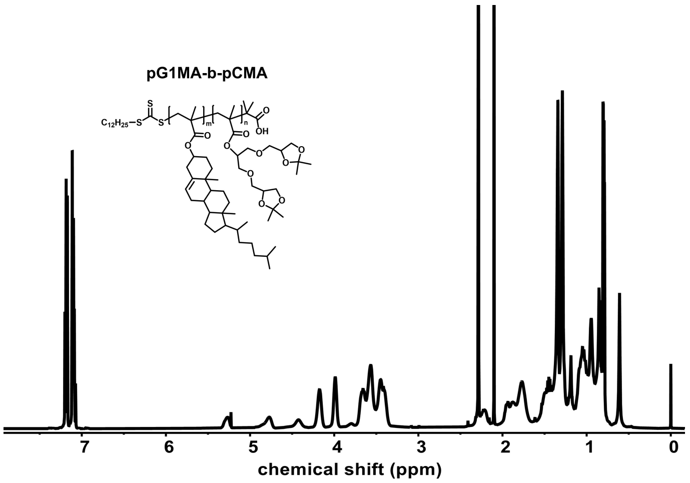

Figure S4. ^{1}H NMR spectrum of the pG1MA-b-pCMA block copolymer.

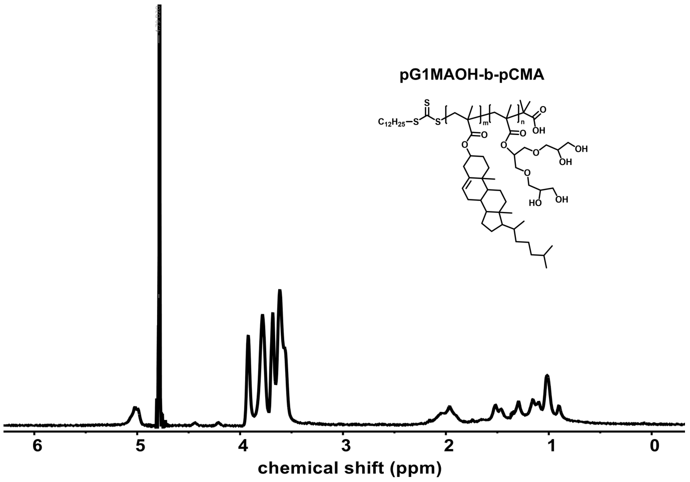

Figure S5. ^{1}H NMR spectrum of the pG1MAOH-b-pCMA polymeric amphiphiles.

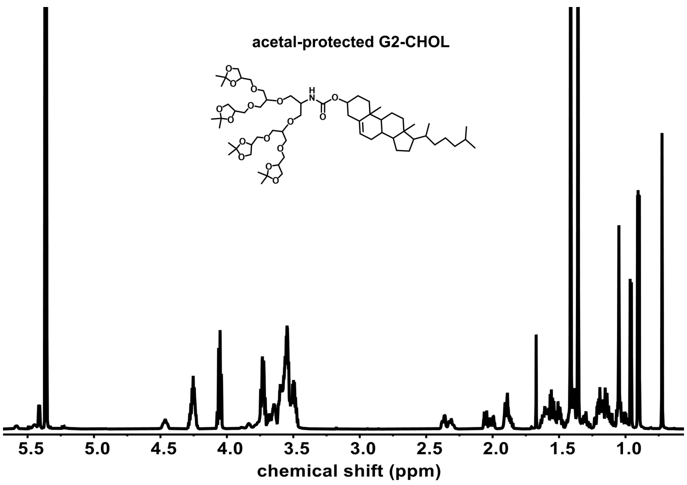

Figure S6. ^{1}H NMR spectrum of the acetal-protected G2-CHOL.

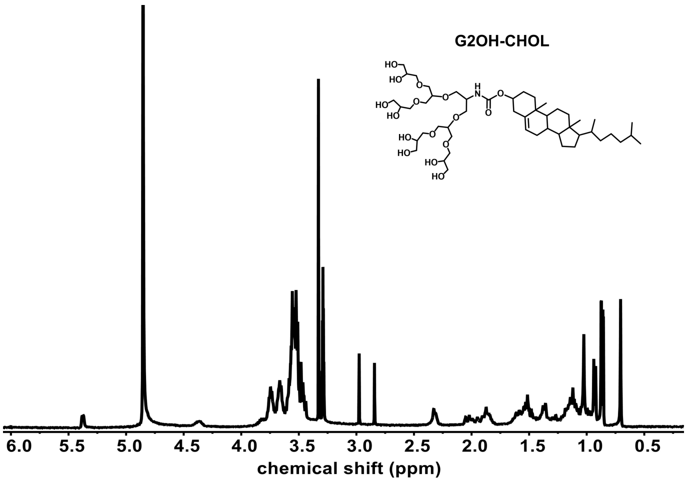

Figure S7. ^{1}H NMR spectrum of the G2OH-CHOL monomeric amphiphiles.

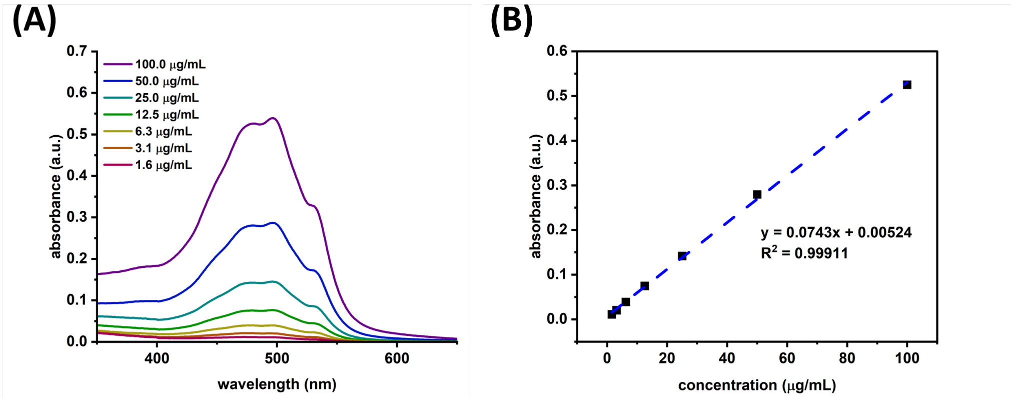

Figure S8. (A) UV/Vis measurements at different concentrations of DOX free base, and (B) calibration curve obtained at 488 nm.

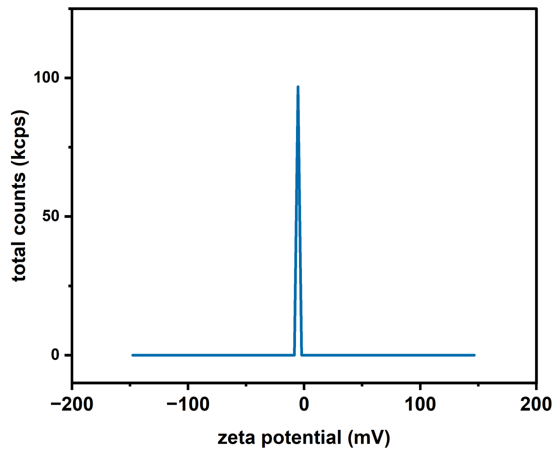

Figure S9. Zeta potential of pG1MAOH-b-pCMA polymeric micelles.

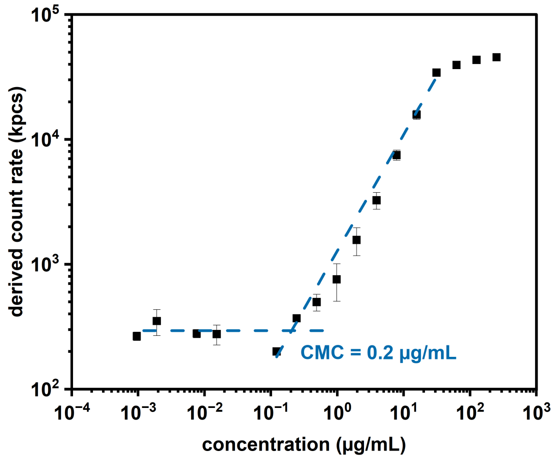

Figure S10. CMC determination of pG1MAOH-b-pCMA polymeric micelles.

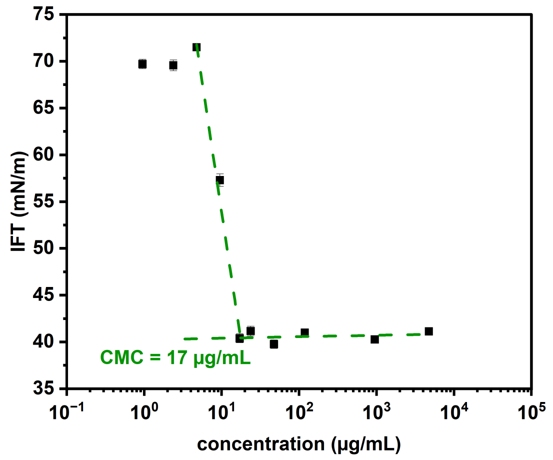

Figure S11. CMC determination of G2OH-CHOL monomeric micelles.

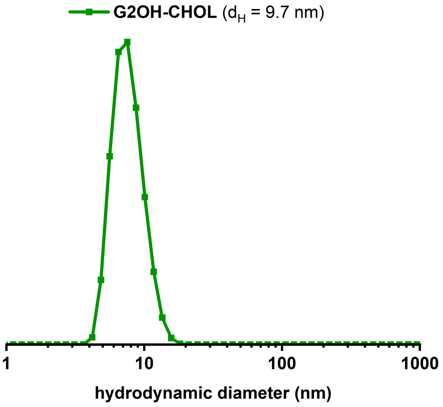

Figure S12. Size distributions (% by volume) from DLS for the G2OH-CHOL monomeric micelles.

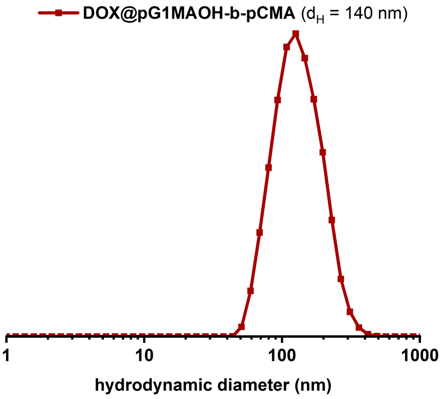

Figure S13. Size distributions (% by volume) from DLS for the DOX@pG1MAOH-b-pCMA.

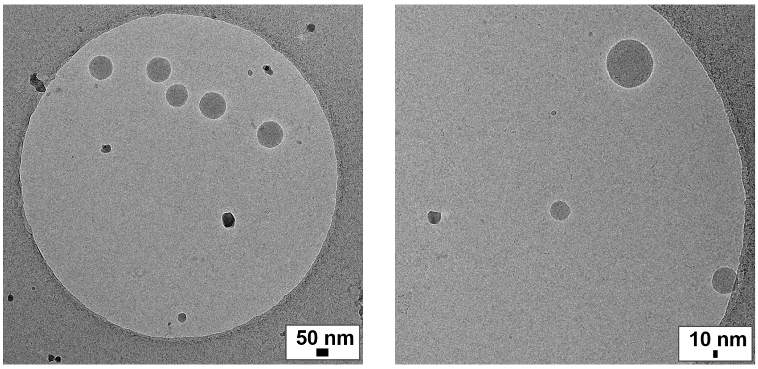

Figure S14. Cryo-TEM micrographs of DOX-loaded polymeric micelles at 28,000× magnification (left) and 45,000× magnification (right).

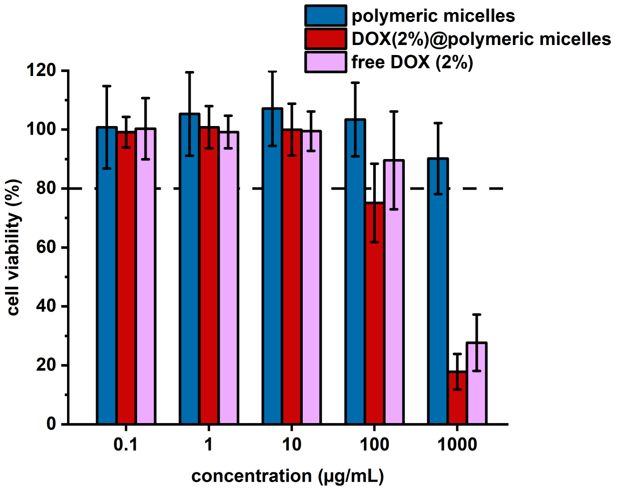

Figure S15. Cell viability of A549 cells treated with polymeric micelles (blue), DOX-loaded polymeric micelles (red), and free DOX (pink). Each bar represents the mean of three independent experiments with standard deviation.

Disclaimer/Publisher's Note: The statements, opinions and data contained in all publications are solely those of the individual author(s) and contributor(s) and not of MDPI and/or the editor(s). MDPI and/or the editor(s) disclaim responsibility for any injury to people or property resulting from any ideas, methods, instructions or products referred to in the content.
